# Sustainable and Inexpensive Polydimethylsiloxane Sponges for Daytime Radiative Cooling

**DOI:** 10.1002/advs.202102502

**Published:** 2021-10-20

**Authors:** Lyu Zhou, Jacob Rada, Huafan Zhang, Haomin Song, Seyededriss Mirniaharikandi, Boon S. Ooi, Qiaoqiang Gan

**Affiliations:** ^1^ Department of Electrical Engineering The State University of New York at Buffalo Buffalo NY 14260 USA; ^2^ Photonics Lab King Abdullah University of Science and Technology Thuwal 23955‐6900 Saudi Arabia

**Keywords:** building envelope, radiative cooling, sustainability, thermal management

## Abstract

Radiative cooling is an emerging cooling technology that can passively release heat to the environment. To obtain a subambient cooling effect during the daytime, chemically engineered structural materials are widely explored to simultaneously reject sunlight and preserve strong thermal emission. However, many previously reported fabrication processes involve hazardous chemicals, which can hinder a material's ability to be mass produced. In order to eliminate the hazardous chemicals used in the fabrication of previous works, this article reports a white polydimethylsiloxane (PDMS) sponge fabricated by a sustainable process using microsugar templates. By substituting the chemicals for sugar, the manufacturing procedure produces zero toxic waste and can also be endlessly recycled via methods widely used in the sugar industry. The obtained porous PDMS exhibits strong visible scattering and thermal emission, resulting in an efficient temperature reduction of 4.6 °C and cooling power of 43 W m^−2^ under direct solar irradiation. In addition, due to the air‐filled voids within the PDMS sponge, its thermal conductivity remains low at 0.06 W (m K)^−1^. This unique combination of radiative cooling and thermal insulation properties can efficiently suppress the heat exchange with the solar‐heated rooftop or the environment, representing a promising future for new energy‐efficient building envelope material.

## Graphical abstract

This work presents a porous polydimethylsiloxane (PDMS) sponge fabricated using a sustainable and inexpensive sugar‐template casting method. The obtained porous PDMS sponge exhibits efficient radiative cooling performance and excellent thermal isolation. This combined feature can suppress the heat exchange from warm outdoor environment, indicating its application for future energy‐efficient building envelope materials.

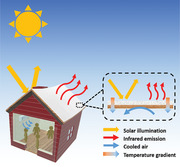

## 1 Introduction

Cooling is a significant end use of energy and a major driver of peak electricity demand. As of 2020, the world consumed up to 392 billion kWh of electricity to cool the interior of commercial and residential buildings.^[^ ^1^ ^]^ However, conventional cooling systems, such as air conditioners, have a very low cooling efficiency, and produce greenhouse gases. These greenhouse emissions will warm the earth and exacerbate the demand for cooling, resulting in a negative feedback loop where the cooling system itself become the source of heat.^[^ ^2^ ^]^ In addition, due to increasing urbanization, the temperature rises much faster in city centers than rural areas (known as the urban heat island (UHI) effect^[^ ^3^ ^]^). The consequences caused by greenhouse effect and UHI effect pose great challenges to cooling. Because of these aforementioned issues, new cooling technologies that are more sustainable and effective are highly desired.

Passive radiative cooling (PRC), an electricity‐free cooling technology that has been exploited for operation during the nighttime,^[^ ^4^, ^5^ ^]^ received renewed interest due to its passive nature and potential to operate during the daytime.^[^ ^6^ ^]^ It is considered as a sustainable alternative to conventional cooling technologies by exploiting the balance of radiative heat flow between terrestrial objects and the outer space. To realize daytime cooling, two general criteria have to be met: 1) in the solar wavelength range (0.3–2.5 µm), a PRC surface must strongly reflect/diffract solar irradiance to minimize solar heating, while 2) in the long‐wavelength infrared range (LWIR, specifically 8–13 µm), a PRC surface spontaneously emits thermal radiation into the universe through a clean sky. Over the past years, extensive research efforts have been devoted to the development of more efficient spectrally selective PRC materials,^[^ ^7^, ^8^, ^9^, ^10^, ^11^, ^12^, ^13^, ^14^, ^15^, ^16^, ^17^, ^18^, ^19^, ^20^, ^21^, ^22^, ^23^, ^24^, ^25^ ^]^ with an averaged cooling power of ≈100 W m^−2^.^[^ ^26^, ^27^, ^28^, ^29^, ^30^ ^]^ More recently, with an optimized system architecture and solar/thermal spectral selective mirrors, a local cooling power density of ≈280 W m^−2^ was demonstrated experimentally in a vertically aligned double‐side emission system.^[^ ^24^ ^]^ This passive cooling capability represents an attractive new candidate for future building envelop materials.^[^ ^31^ ^]^ Along this research direction, several designs have been reported including cooling wood,^[^ ^11^ ^]^ white paints,^[^ ^22^ ^]^ polymer coating,^[^ ^9^ ^]^ and plastics.^[^ ^32^ ^]^ In order to obtain the desired high solar reflectance, nanoparticles^[^ ^8^ ^]^ or boiling special solvent solutions^[^ ^9^ ^]^ were employed to produce nano/micro pores that strongly scatter sunlight. However, many of the key gas‐phase solvents or micrometer‐sized particles involved in these processes are hazardous to humans, or are harmful to the environment.^[^ ^33^ ^]^

**Figure 1:**
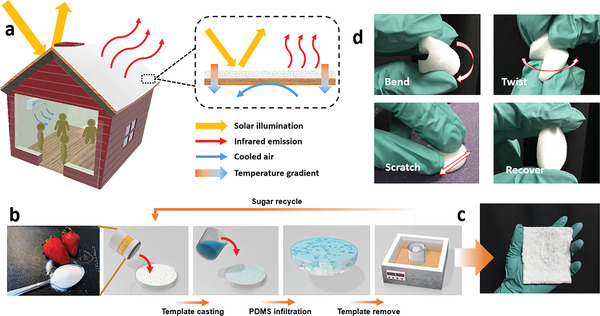
a) Schematic illustration of a radiative cooling roof integrated with a residential building. b) Schematic illustration of the fabrication process for porous PDMS sponges. c) A photo of an as‐prepared porous PDMS sponge. d) Photos showing the flexibility of the PDMS sponge.

In this work, we propose a sustainable and inexpensive template‐casting method to fabricate a porous polydimethylsiloxane (PDMS) sponge for PRC.^[^ ^34^ ^]^ The entire process only involves sugar particles (or salt microparticles), which is preferred for industrial mass production at very low costs. The obtained porous PDMS sponge exhibits strong scattering in the visible range, and strong thermal emission in the infrared range, meeting the criteria for efficient daytime PRC. In particular, one of the envisioned key applications for PRC is to provide supplementary cooling to commercial and residential buildings.^[^ ^22^, ^35^ ^]^ As illustrated in **Figure** **1a**, an ideal roofing material is capable of rejecting the incoming solar illumination and dissipating the heat via radiation, reducing the cooling load of the building. However, since the ambient temperature in tropical areas can go beyond 40 °C (where active cooling is more demanding), radiative cooling alone is not sufficient to meet the target temperature for convenient living environment. Excellent thermal isolation features are also desired to prevent thermal conduction from the warmer environment into the living environment. We will show that the proposed PDMS sponge can also meet this extra criterion of thermal insulation. Our experiment demonstrated a cooling power of 43.17 W m^−2^ and a temperature drop of 4.6 °C under 1 sun illumination. By placing the PDMS sponge on the roof of foam‐based chambers under direct sunlight (to mimic the residential houses), our material can maintain a low indoor temperature due to its superior solar scattering and thermal isolation features. The combination of both a sustainable fabrication process, and radiative cooling capabilities, will allow this material to be utilized as a promising energy‐efficient cool roofing material.

## 2 Results

### 2.1 Fabrication of Porous PDMS Using Sugar Microparticle Templates

Although various porous polymer films have been reported to realize remarkable radiative cooling performance, several key processes contain hazardous chemicals that are not desired in mass production (e.g., acetone can induce skin irritation and many other negative effects to human with excessive inhalation/exposure^[^ ^36^ ^]^). Recently, sustainable processes were reported to realize porous PDMS films using microdroplets of water.^[^ ^21^, ^37^, ^38^, ^39^ ^]^ By mixing water and PDMS emulsion through different strategies (e.g., plasma treatment,^[^ ^37^ ^]^ ultrasonication,^[^ ^37^, ^38^ ^]^ vortex mixer,^[^ ^39^ ^]^ or metal catalysts^[^ ^21^ ^]^), uniform porous PDMS films were obtained that can realize switchable radiative cooling features (e.g., ref. ^[^ ^21^ ^]^). Here we report an alternative scalable and sustainable fabrication strategy by employing sugar sacrificial templates to realize porous PDMS sponge for radiative cooling. The general fabrication procedure is shown in Figure 1b: fine granulated crystal sugar powders (Domino with the particle size of 100–600 µm) were first poured into the mold to form the template. The premixed PDMS precursor (which is considered as a safe chemical material^[^ ^40^ ^]^) was then added into the mold. The mixtures were settled for at least 8 h to allow PDMS precursor fully infiltrated into the pores in the sugar template. After curing, the PDMS was subjected to a water bath (at the temperature of 60 °C) in order to dissolve the sugar. The PDMS film was then air dried at 80 °C for half an hour to complete the fabrication process. As a result, a white PDMS sponge was obtained, as shown in Figure 1c. Importantly, the sugar used in the fabrication process can be recycled via simple crystallization method that is widely used in sugar industry.^[^ ^41^ ^]^ The entire manufacturing has zero toxic chemical residue, indicating the sustainability of this process. Importantly, the solution‐based mixture of PDMS and sugar templates are particularly suitable for industrial roll‐to‐roll painting processes for mass production that can readily compete with commercial roofing materials. In addition, as one can see from Figure 1d, the sponge recovered instantly after bending, twisting or scratching on a sand paper, indicating its excellent flexibility and elasticity (see Video S1 in the Supporting Information). Next, we will characterize its optical and thermal properties to demonstrate its spectral selectivity required by PRC.

### 2.2 Optical Characterization of the Porous PDMS Sponge

**Figure 2:**
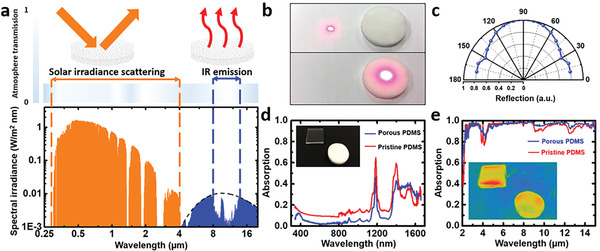
a) Modeled solar irradiation spectrum and atmospheric radiation spectrum. b) Photos of scattering spot on a white paper and a porous PDMS when illuminated by red lasers (650 nm). c) Measured angle dependent reflection of porous PDMS at 500 nm. d,e) Measured absorption spectrum of porous PDMS (blue curve) and pristine PDMS (red curve). Inset figures show the photo and IR image of two PDMS, respectively. Both samples have a thickness of 3 mm.

Daytime radiative cooling actually relies on spectrum selection, i.e., one needs to 1) minimize solar absorption and 2) maximize the thermal emission power simultaneously. Therefore, the major requirements include: the object must strongly reflect solar wavelengths (i.e., the orange spectrum in **Figure** **2a**) and highly emit thermal radiation in atmospheric transparency window (i.e., the gap of the blue spectrum in Figure 2a). In recent years, various advanced optical and thermal materials have been reported to optimize this spectral selectivity.^[^ ^6^, ^8^, ^9^ ^]^ In term of the price‐performance ratio, inexpensive PDMS films stand out due to their unique optical and thermal features.^[^ ^14^ ^]^ However, since PDMS is visibly transparent, a strongly reflective substrate (e.g., silver) is usually required to minimize solar absorption by the substrate under the thermal emitter, resulting in a cost barrier for large‐scale applications. Therefore, the obtained porous PDMS sponge in Figure 1 is of interest to further overcome this cost barrier. Due to the intrinsic porosity, the PDMS sponge is able to strongly scatter the incident light. To directly show its scattering feature, in Figure 2b, we employed a red laser to illuminate a regular white paper (the upper panel) and the PDMS sponge (the lower panel). We also characterized its angle dependent reflectivity (see the Experimental Section for details) to quantify its scattering feature. As shown in Figure 2c, the reflectivity of the PDMS sponge is strong over a wide spatial angle range, explaining the white color of the sample under regular white light illumination.

To further investigate the solar heating effect of the porous PDMS sponge, we measured its optical absorption spectrum using an integration sphere spectrometer (see the Experimental Section for details), and plotted results in Figures 2d,e, respectively. One can see that in the wavelength range from 350 to 750 nm, the absorption of the porous PDMS sponge (*A*_solar_porous_ = 0.07) is much smaller than the absorption of the pristine PDMS film (*A*_solar_pristine_ = 0.12). The significant suppression of solar absorption can be attributed to the diffused reflection by pores. According to the optical property of PDMS, a relatively large refractive index (n) change occurs at the air/PDMS interface (*n*_PDMS_ = 1.38 for visible wavelengths as shown in Figure S1 in the Supporting Information), leading to reflection as the incident light interacts with the interface of each pore and, therefore, meeting the aforementioned criteria (1) of the required spectral selectivity. Regarding criteria (2), both the pristine and porous PDMS samples show strong thermal emission in infrared wavelength range, as shown in Figure 2e. The measured absorption spectrum also indicates that in the atmospheric transparency window (i.e., 8–13 µm), the porous PDMS sponge has an average emissivity $\bar{\varepsilon }$_porous_ of 0.96, slightly higher than the pristine PDMS sample ($\bar{\varepsilon }$_pristine_ = 0.94). As a result, the fabricated porous PDMS sponge shows obvious improvement in spectral selectivity, especially in solar absorption suppression. Intriguingly, considering different sugar (or salt) products available in the market, different pore sizes can be obtained following the same fabrication procedure to produce different PDMS sponges. Next, we will characterize optical and thermal spectral features of porous structures with different porosities.

### 2.3 Morphology Dependence

**Figure 3:**
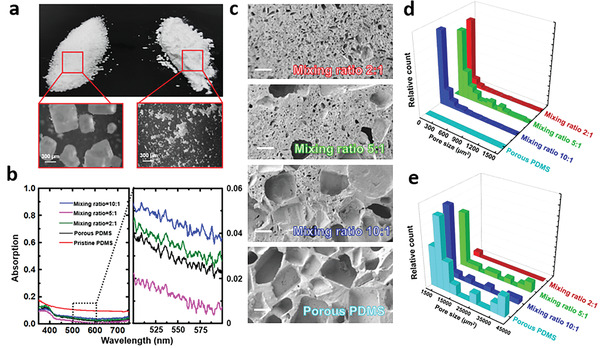
a) Photos (upper) and microscope images (bottom) of two different sugar particles. b) Measured reflection spectra of the four different porous PDMS and a pristine PDMS in the visible wavelength range. c) SEM images of four porous PDMS samples with different pore distribution. The scale bars in SEM images are 150 µm. d,e) Extracted pore size distributions from (c).

To reveal the structural dependence of porous PDMS sponges, we fabricated several samples with different morphologies. Hierarchical porous structures were formed using different sugar templates by mixing granulated crystal sugar (Domino) with ultrafine sugar powders (Good & Gather) at different weight ratios of 10:1, 5:1, and 2:1. **Figure** **3a** shows photos and microscope images of the sugar particles: The size of the crystal sugar particle is 100–600 µm, while the size of the sugar powder is smaller than 10 µm. We then measured the visible spectra of porous PDMS samples fabricated by these sugar templates and plotted the results in Figure 3b. For comparison, the spectra of the previously fabricated porous PDMS and a pristine PDMS shown in Figure 2 are also included in this figure. One can see that all porous PDMS samples exhibit smaller average absorption (<0.06) compared to the pristine PDMS sample (≈0.12). Due to the varied porous structure, the PDMS sponge fabricated with the mixing ratio of 5:1 shows the lowest absorption (0.01) over the measured visible spectrum. To further reveal the structural mechanism of the suppressed absorption, we characterized scanning electron microscope (SEM) images of these samples, as shown in Figure 3c. Using these SEM images, pore size distributions were extracted and plotted in Figures 3d,e. One can see the optimized sample (with the mixing ratio of 5:1) contains two peaks centered at ≈100 µm^2^ and 1600 µm^2^, respectively. Compared with the porous PDMS sponges fabricated using large sugar particles only, these smaller pores slightly improved the optical scattering feature that is desired for solar absorption suppression.^[^ ^9^ ^]^ We attribute this enhanced scattering to the wider distribution of pore size introduced by the mixed sugar template, which can introduce stronger diffuse reflection^[^ ^42^ ^]^ due to the randomly orientated interfaces inside the porous structure. It should be noted that it is more difficult to dissolve ultrafine sugar powder in the PDMS system due to the small pore size. The water diffusion rate into the hierarchical porous structures is much slower than that formed by large sugar particles. Considering the manufacturing easiness and performance‐cost ratio, we then still used porous PDMS produced in Figure 1 for outdoor experiments.

### 2.4 Outdoor Test and Analysis

**Figure 4:**
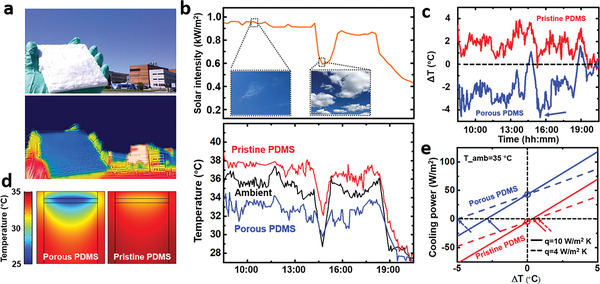
a) Photo and IR image of a porous PDMS film exposed to the sky at Buffalo, NY. b) Continuous outdoor temperature measurement of porous PDMS and pristine PDMS, and the measured weather conditions (August 27, 2020). The black, red and blue curve represents ambient temperature, the temperature of pristine PDMS and porous PDMS, respectively. c) Extracted temperature difference achieved by pristine PDMS and porous PDMS from (b). d) COMSOL modeling results of two PDMS emitters at ambient temperature of 35 °C. e) Estimated cooling power of pristine PDMS and porous PDMS as a function of temperature difference. Dashed and solid curve represent the situations with *q* = 4 W m^−2^ K^−1^ (dashed curve) and *q* = 10 W m^−2^ K^−1^ (solid curve), respectively.

In order to reveal the cooling capability of these sugar‐template‐produced porous PDMS samples, we performed a series of outdoor tests at Buffalo, NY (Figure S2, Supporting Information). As shown in **Figure** **4a**, the surface temperature of a sky‐facing porous PDMS film is obviously lower than the temperature of a building under direct sunlight illumination. In order to characterize the cooling effect quantitatively, two experimental setups were built using a porous PDMS sponge and a pristine PDMS film as thermal emitters, respectively (see experimental details in Note S1 in the Supporting Information). Two K‐type thermal probes were employed to measure their surface temperature (*T*_PDMS_). As shown in Figure 4b, on a sunny day on August 27, 2020, we performed a continuous outdoor test over 12 h and monitored the surface temperature of the two thermal emitters in these two systems (see more results in Figures S3 and S4 in the Supporting Information). The incident solar intensity is shown in the upper panel while the surface temperatures of those two thermal emitters are plotted in the lower panel. One can see that the temperature of the pristine PDMS sample is always higher than the ambient due to the direct solar heating effect. In contrast, the porous PDMS sponge realized continuous daytime radiative cooling with no need of sunlight shelter. To further reveal the cooling performance, the temperature differences between the PDMS emitters and ambient are plotted in Figure 4c, showing that the porous PDMS sponge maintains a temperature reduction of 3.2 ± 1.0 °C below the ambient, while the pristine PDMS film can only realize a temperature that is 2.0 ± 1.4 °C higher than the ambient. In particular, the best temperature reduction of 4.6 °C was obtained around 15:30 (see the blue arrow in the lower panel in Figure 4c) due to the clean sky. In contrast, the obvious dip of temperature was observed around 14:30 because of the thick cloud blocking the sunlight (see the right inset in the upper panel of Figure 4b). This observation clearly indicates the weather dependence of radiative cooling technology on solar intensity, humidity and sky clearness, etc.

To further interpret the experimental observation, we then employed COMSOL package to model the radiative cooling effect on porous and pristine PDMS films under direct sunlight illumination (see Note S2 and Figure S5 in the Supporting Information). As shown in Figure 4d, the left architecture with a porous PDMS emitter shows obvious temperature reduction of 7.7 °C, while the right architect with the pristine PDMS emitter shows a temperature that is 2.9 °C lower than the ambient (*T*_amb_ = 35 °C). The slightly different cooling performance should be attributed to the ideal atmospheric transmittance used in the numerical model (see Note S2 in the Supporting Information for details). Intriguingly, building upon these numerical modeling results, one will be able to estimate the corresponding cooling power of the porous PDMS emitter.

Due to the poor thermal conductivity of porous PDMS, it is difficult for us to employ feedback control method^[^ ^6^, ^8^ ^]^ to monitor the cooling power in the outdoor experiments. Instead, we can calculate the net cooling powers, *P*_net_, of the two thermal emitters as the function of the temperature difference (Δ*T* = *T*_PDMS_ *− T*_amb_) using Equation (1)

(1) $$\begin{array}{c} P_{\mathrm{net}}=P_{\mathrm{rad}}\left(T_{\mathrm{PDMS}}\right)-P_{\mathrm{atm}}\left(T_{\mathrm{amb}}\right)-P_{\mathrm{sun}}-P_{\mathrm{nonrad}}\left(T_{\mathrm{PDMS}},T_{\mathrm{amb}}\right) \end{array}$$

here *P*_rad_(*T*_PDMS_) is the emitted radiation power from the PDMS emitter, *P*_atm_(*T*_amb_) is the absorbed atmospheric radiation power, *P*_sun_ is the absorbed solar irradiance and *P*_nonrad_(*T*_PDMS_, *T*_amb_) is the nonradiative heat transfer between the PDMS emitter and environment (Note S3, Supporting Information). The results were estimated using the spectra shown in Figure 2d,e and the atmospheric transmittance was modeled using MODTRAN. As shown in Figure 4e, we plotted the cooling powers of both porous and pristine PDMS samples with different nonradiative heat coefficients q of 4 W m^−2^ K^−1^ and 10 W m^−2^ K^−1^, respectively (corresponding to two different convection conditions depending on natural wind). Under a solar irradiance of 980 W m^−2^, the cooling power (indicated by two circles) of the porous PDMS sponge reached 43.17 W m^−2^, which was much higher than that of the pristine PDMS film (i.e., with a heating power of 4.69 W m^−2^). The corresponding temperature reductions of the porous PDMS sponge under these two different conditions are −5 and −2.8 °C, respectively (as indicated by two blue arrows in Figure 4e), agreeing with the temperature fluctuation range observed in Figures 4b,c. Importantly, this type of porous PDMS sponge is particularly attractive to serve as an energy saving building envelope material, as will be discussed in the next paragraph.

**Figure 5:**
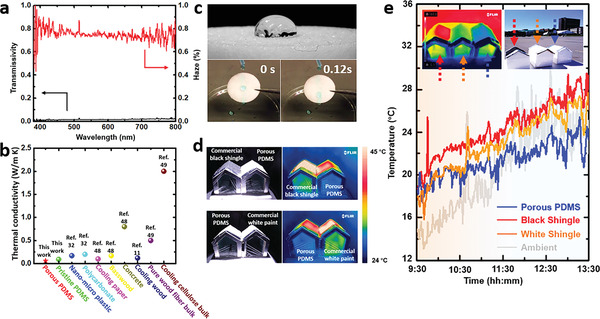
a) Measured transmissivity spectrum and haze of a 3 mm porous PDMS sponge. b) Measured thermal conductivity of the PDMS sponge and its comparison with other materials reported by previous literature. c) Photos to demonstrate the hydrophobicity of the PDMS sponge. d) Photos and infrared images of three different house model with roofs made of black asphalt shingle, asphalt shingle coated with white paint and porous PDMS sponges. e) An outdoor test showing radiative cooling and thermal isolation features of the PDMS sponge roof, compared with a commercial black shingle roof and a white‐painted shingle roof. Inset: A photo of the outdoor test and the thermal image of the model houses under direct sun light illumination.

Typical roofing materials include shingles, metals, plastics and concretes.^[^ ^43^ ^]^ However, due to the dark color of many roofing materials, the incident solar energy resulted in heating to the roofs of residential houses/buildings, especially over summertime.^[^ ^44^ ^]^ It was estimated by Department of Energy (US) that a white color roof can simply reduce the electricity consumption of a typical building by 10–15%.^[^ ^45^ ^]^ Here we will further reveal the potential of the porous PDMS sponge to serve as a building envelop to further reduce the electricity consumption for air‐conditioning. As shown in **Figure** **5a**, a porous PDMS sponge with thickness of 3 mm exhibits extremely low optical transmittance (≈1%) and high optical haze of ≈75% (i.e., the amount of light that is subject to wide angle scattering). These results lead to a unique antiglare feature that is highly desired in future energy‐efficient building materials.^[^ ^46^ ^]^ In addition, PDMS was proved stable under full solar exposure.^[^ ^47^ ^]^ As shown in Figure S6 (Supporting Information), we performed aging tests up to 300 h under solar illumination for a porous PDMS sample and observed little difference in its reflection property. Moreover, as a roofing material, it is normally desired to have a high thermal resistance to protect the interior space from the constantly varied environment. As shown in Figure 5b, we characterized the thermal conductivity of the porous PDMS sponge and compared the measured value with previously reported radiative cooling materials. Interestingly, our PDMS sponge shows a thermal conductivity of 0.06 W (m K)^−1^, which is the lowest value among recently reported radiative cooling materials and is suitable for building envelope.^[^ ^11^, ^29^, ^48^, ^49^ ^]^

To demonstrate its potential to serve as a building envelope, we first revealed that the PDMS sponge is hydrophobic, indicating its capability to block rain which is one of the most important features for building envelop materials. As shown in the upper panel in Figure 5c, the water contact angle is 106.0° (Figure S7, Supporting Information). When we introduced water droplets on its surface (lower panel in Figure 5c), those droplets will slide down immediately with no residuals on the porous PDMS surfaces, which is promising to realize self‐cleaning features.^[^ ^50^ ^]^ We then developed three model houses using foams and covered by a black commercial asphalt shingle (Royal Sovereign Charcoal Algae Resistant 3‐Tab Roofing Shingles, GAF), an asphalt shingle coated with commercial white paints (2X Ultra cover, white paint, Rust‐Oleum) (See reflection spectrum in Figure S8, Supporting Information), and our porous PDMS sponge. A window was opened on the side wall in each house model and sealed by an infrared‐transparent polyethylene film. In this case, the interior temperature can be directly observed by an infrared camera (FLIR one Pro., FLIR LLC). In this experiment, we placed different models under a solar simulator and monitored their inside temperatures. As shown in Figure 5d, due to the stronger visible absorption, a black asphalt shingle shows significantly higher surface temperature (i.e., 66.8 °C) than the porous PDMS sponge roof (i.e., 43.7 °C). Considering the thermal conductivities of these two materials, the heat flow can be estimated using Fourier's law, i.e., the heat flow through the black asphalt shingle is 760 W m^−2^, much higher than that of the PDMS sponge (i.e., 76 W m^−2^) (see Note S4 in the Supporting Information for more details). On the other hand, comparing the porous PDMS sponge roof with the white‐painted asphalt shingle roof (i.e., Figure 5d), one can see their surface temperature is similar under 1 sun illumination (i.e., ≈43 °C). However, the interior temperature under the porous PDMS roof is 3.7 °C lower than that under the white painted asphalt shingle roof, mainly due to the suppressed thermal conductivity of the PDMS sponge.

Finally, we performed the outdoor test on May 17, 2021 at Buffalo (with a relatively clean sky and windy weather, see the inset photo in Figure 5e). Under a regular sun illumination (with the intensity of ≈900 W m^−2^), the surface temperature of the commercial shingle is obviously higher than that on the porous PDMS sponge (see the inset for the thermal image). The measured ambient temperature (the gray curve) and the temperatures inside the three model houses are plotted in Figure 5e. One can see that the temperature in the model with a commercial shingle roof (red curve) and a white‐painted shingle roof (orange curve) is always higher than the ambient temperature. It should be noted that the surface temperature of the commercial shingle was not very high due to the rapid thermal convection introduced by relatively frequent wind on that day. In contrast, the temperature in the model with the PDMS sponge (blue curve) is ≈2 °C below the ambient (see 11:45‐13:30 on the blue curve) due to its radiative cooling feature. In addition, when the ambient is relatively cool (or cold) in the early morning (e.g., from 9:30 to 10:45), the interior temperature in the model house with porous PDMS roof is warmer due to the great thermal isolation feature. This outdoor experiment demonstrates the potential of the proposed PDMS sponge to serve as an energy‐efficient building envelope material for better thermal management.

## 3 Conclusion

In conclusion, we reported a porous PDMS sponge for efficient daytime radiative cooling. This material is fabricated using inexpensive sugar templates, and completely avoids the reported hazardous chemicals. The sustainable manufacturing procedure can recycle sugar powders through industrial sugar crystallization and production processes. The obtained porous PDMS shows simultaneous suppressed solar absorption and strong thermal emission, enabling efficient daytime radiative cooling under direct solar illumination. In addition to its radiative cooling capabilities, other beneficial characteristics of the porous PDMS sponge, i.e., strong mechanical strength and good thermal insulation, make it an ideal candidate for energy‐efficient building envelope materials. Our preliminary experiment demonstrates that the porous PDMS sponge roof can reduce the heat leakage to the space under the roof by ≈90% compared with a commercial asphalt shingle product. This unique combination of radiative cooling and thermal insulation properties can efficiently suppress the heat exchange with the direct sun light or the environment, which will allow for the development of new energy‐efficient building envelope materials, and can also be used in improving HVAC systems.

## 4 Experimental Section

### Materials

Polydimethylsiloxane (Sylgard 184) was purchased from Dow Corning. Commercial sugar powders used in the fabrication were Domino (i.e., Domino premium, pure cane, granulated sugar, with large particle size), and Good & Gather powdered sugar (with small particle size). These materials are all inexpensive. The equivalent density of porous PDMS is estimated by measuring its weight and volume. Considering the following parameters for the porous PDMS disk sample (i.e., diameter = 25.4 mm; average thickness = 5 mm; and weight = 1.28 g), its equivalent density is ≈505 kg m^−3^. By taking the retail price of PDMS into account, the estimated cost of a 3 mm thick porous PDMS film is ≈$171 m^−2^ under the laboratory conditions.

### Optical Characterization

The visible reflection spectrum was measured using integrating sphere spectrometers and a commercial spectrometer (Agilent Cary 7000 Universal measurement spectrophotometer). For the ultraviolet‐to‐visible range (350–750 nm) and visible‐to‐near infrared range (750–1650 nm), the porous PDMS sample was attached on a PTFE‐based back reflector and the reflection spectrum was measured using a PTFE‐based integrating sphere (Model IS200 Series Integrating Spheres, Thorlabs) coupled with two different spectrometers (Ocean Optics JAZ‐COMBO, Ocean Optics and AvaSpec‐NIR256‐1.7‐TEC, Avantes). For the mid‐infrared range (2–15 µm), the reflection spectrum was measured by a mid‐IR integrating sphere (A562, Bruker) coupled with Bruker VERTTEX 70 FTIR. The angular‐dependent reflection was measured using Agilent Cary 7000.

## Conflict of Interest

The authors declare no conflict of interest.

## Supporting information

Supporting Information

Supplemental Video 1

## Data Availability

The data that support the findings of this study are available from the corresponding author upon reasonable request.
