# Supplementary material for: Sustainable and Inexpensive Polydimethylsiloxane Sponges for Daytime Radiative Cooling
Source: Adv Sci (Weinh). 2021 Oct 20;8(23):2102502. doi: 10.1002/advs.202102502 (PMC8655219; doi:10.1002/advs.202102502)
Supplement: Supplementary file 1 — Supporting Information [file ADVS-8-2102502-s002.pdf]

© 2021 Wiley-VCH GmbH

ADVANCED SCIENCE
Open Access

Supporting Information

for *Adv. Sci.*, DOI: 10.1002/advs.202102502

Sustainable and Inexpensive Polydimethylsiloxane Sponges for Daytime Radiative Cooling

Lyu Zhou[1], Jacob Rada[1], Huafan Zhang[2], Haomin Song[1], Seyededriss Mirniaharikandi[1], Boon S. Ooi[2], Qiaoqiang Gan[1]

Supporting Information

Sustainable and Inexpensive Polydimethylsiloxane Sponges for Daytime Radiative Cooling

Lyu Zhou[1], Jacob Rada[1], Huafan Zhang[2], Haomin Song[1], Seyededriss Mirniaharikandi[1], Boon S. Ooi[2], Qiaoqiang Gan[1]

1 *Department of Electrical Engineering, The State University of New York at Buffalo, Buffalo, NY 14260, USA.*

2 *Photonics Lab, King Abdullah University of Science and Technology, Thuwal 23955-6900, Saudi Arabia.*

Table of contents

Figure S1. The refractive index (n) of PDMS.

Figure S2. The outdoor radiative cooling setup.

Figure S3. Outdoor radiative cooling tests performed at Buffalo on Sep. 14th and Oct. 7th, 2020.

Figure S4. Outdoor radiative cooling tests performed at Buffalo on Sep. 15th, 2020.

Figure S5. CMOSOL modelling results for temperature distributions of PDMS emitters.

Figure S6. The aging test of porous PDMS.

Figure S7. Measured water contact angles of porous and pristine PDMS samples.

Figure S8. Measured reflection spectra of a white-painted shingle and a black commercial shingle.

Note S1. Outdoor radiative cooling tests.

Note S2. CMOSOL modelling of PDMS in a daytime radiative cooling test.

Note S3. Estimation of cooling powers for porous and pristine PDMS emitters

Note S4. Estimation of heat fluxes through different roofing materials

Supporting Video S1: Observation of the flexibility and durability of the proposed porous PDMS sponge

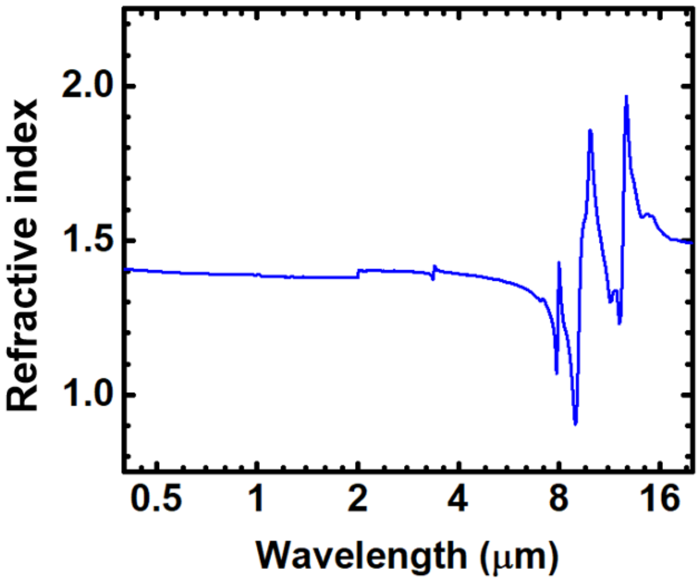

Figure S1. The refractive index (n) of PDMS from ref. [R1-R2].

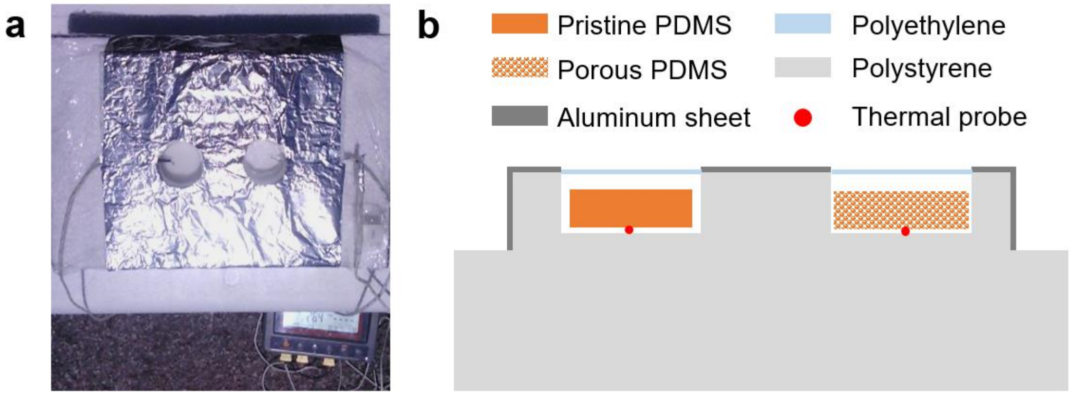

Figure S2. The outdoor radiative cooling setup.

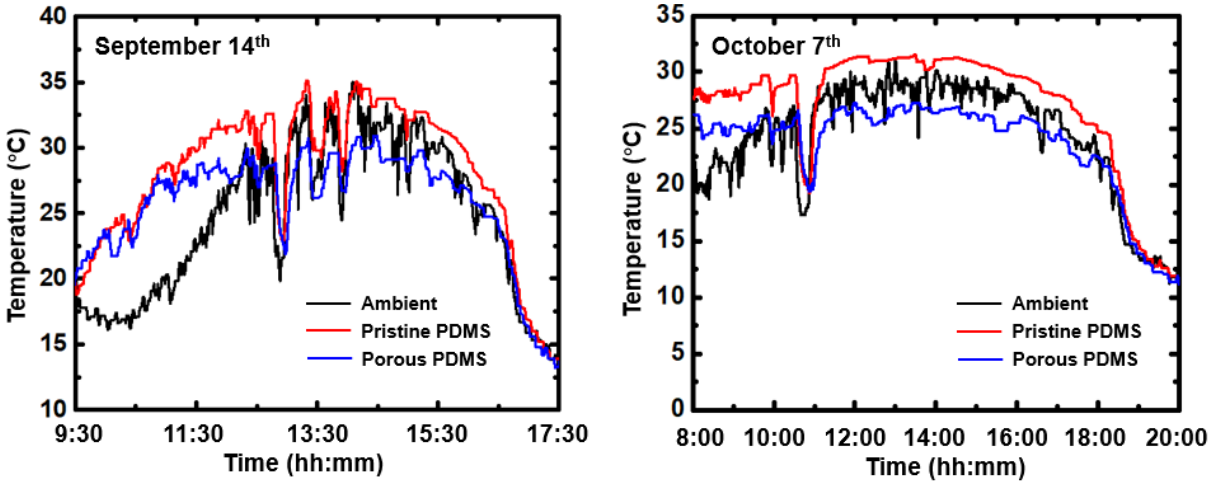

Figure S3. Outdoor radiative cooling tests performed at Buffalo on Sep. 14th and Oct. 7th, 2020. Due to the clear sky condition on these two days, the porous and pristine PDMS films showed similar cooling/heating behavior shown in Figure 4b.

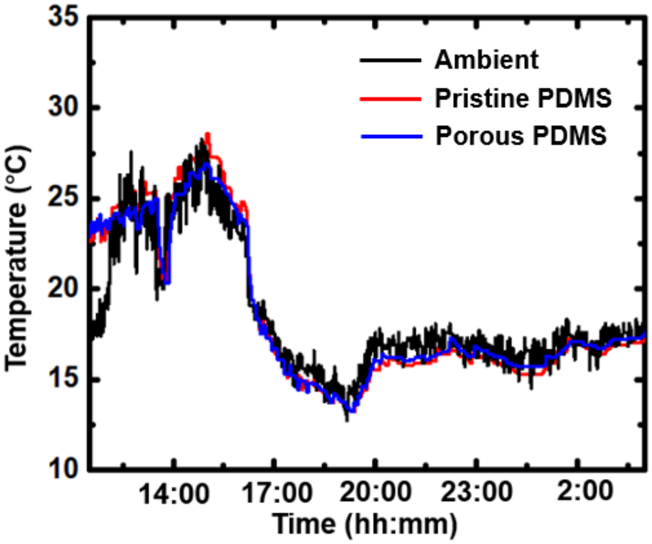

Figure S4. Outdoor radiative cooling tests performed at Buffalo on Sep. 15th, 2020. Due to the cloudy sky condition, the porous and pristine PDMS films showed similar temperature, which is close to the ambient.

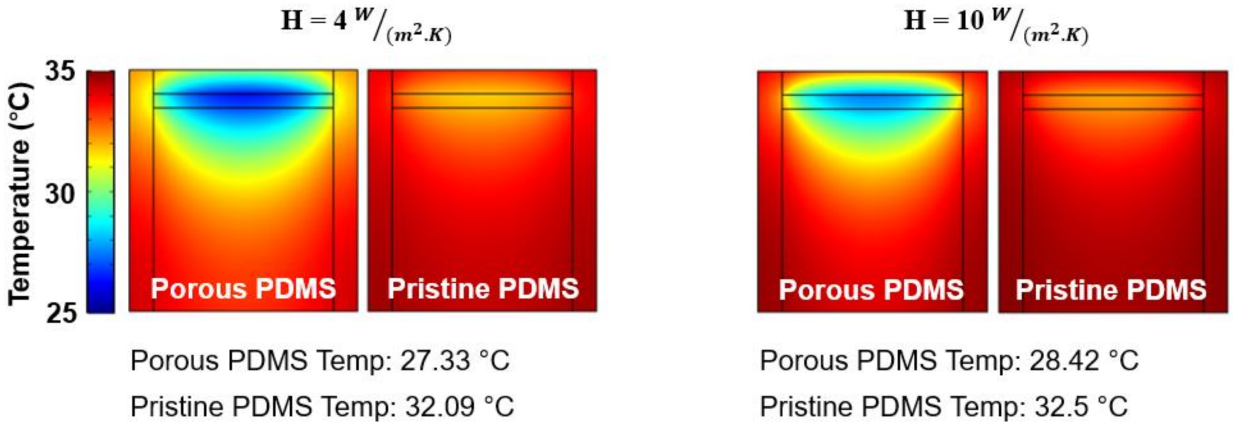

Figure S5. CMOSOL modelling results of PDMS in a daytime radiative cooling test. The ambient temperature was set to 35 °C (close to the measured ambient temperature in Fig. 4b of the main text).

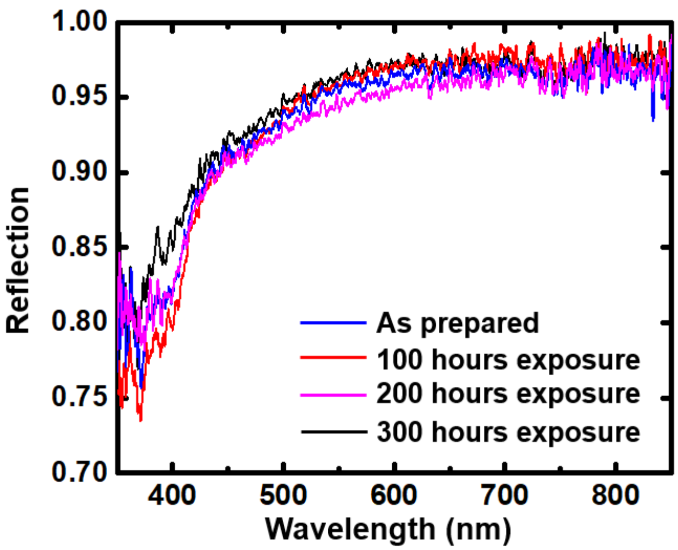

Figure S6. Aging tests of porous PDMS: measured reflection spectrum of porous PDMS under different solar exposure periods.

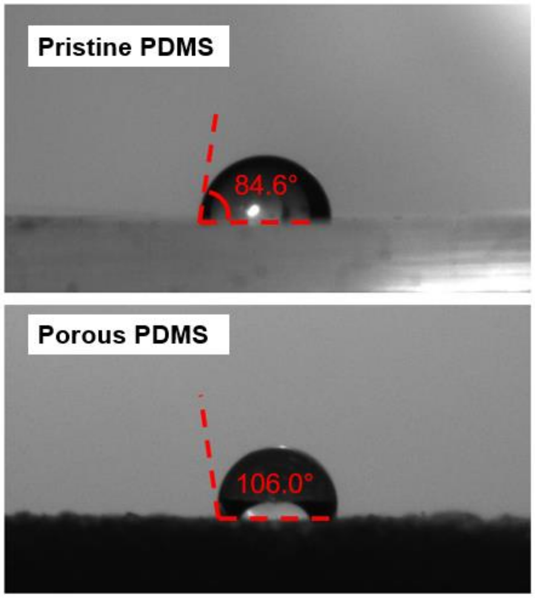

Figure S7. Measured contact angle of a droplet on the pristine PDMS film (the upper panel) and the porous PDMS sponge surface (the lower panel).

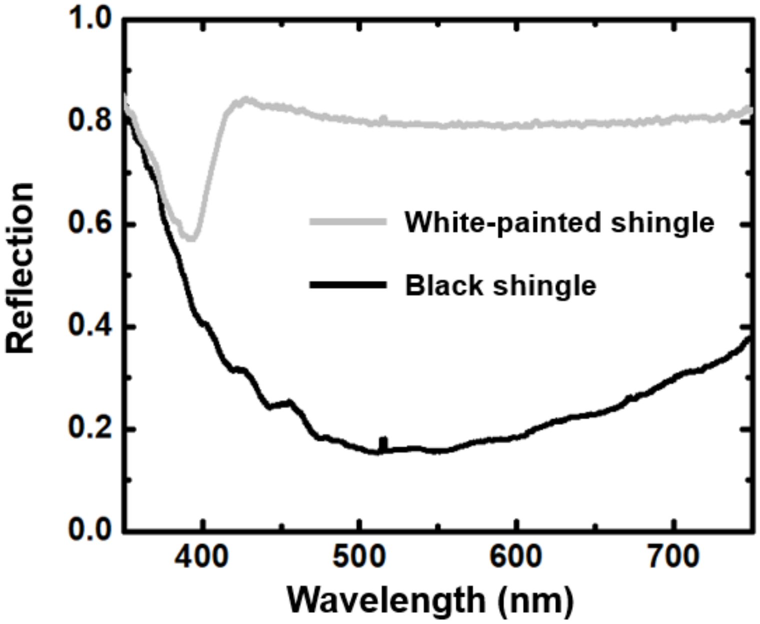

Figure S8. Measured reflection spectra of a white-painted shingle (grey curve) and a black commercial shingle used in Figure 5 (black curve).

Note S1. Outdoor radiative cooling tests

In our outdoor radiative cooling tests, a proof-of-concept experimental setup was built (***Figure. S1***) to simultaneously measure the surface temperature of a porous PDMS sponge and a pristine PDMS film. Both emitters were trimmed into a circular shape with the diameter of 3 cm and the thickness of 5 mm. To minimize the parasitic heat loss, we used polystyrene foam to build the setup and wrapped it with the aluminum tape. During the test, both emitters were placed under direct solar illumination.

Note S2. COMSOL modelling of PDMS in a daytime radiative cooling test.

A theoretical model was developed by coupling the Heat Transfer in Solids interface with the Surface-to-Surface Radiation interface in the software (see an example [R3]). We studied the model in the spectrum range from 0.4-20 μm. In the modelling, the solar irradiance was assumed to 1000 W/m^2 with the diffuse solar irradiance, $I_{solar-diffuse}(\lambda)=0$, and the ambient temperature (T_{amb}) of 35 ℃. The emissivity of atmosphere was assumed to 1 in the range of 2.5-8 μm and 13-20 μm, where the rest of the emissivity was assumed to be 0. The absorptivity of the porous and pristine PDMS films were defined by the spectra measured in Figure 2d and 2e, respectively.

In this simulation, we studied two different convection heat transfer coefficients of q= 4 and 10 $W/(m^2 \cdot K)$ to numerically validate the maximum and minimum steady-state temperature distribution obtained in Figure 4b. The obtained results are plotted in Figure 4d and ***Figure S4***.

Note S3. Estimation of cooling powers for porous and pristine PDMS emitters

The cooling power of PDMS emitter can be estimated using equation 1 in the main text, as described below:

$$P_{net} = P_{rad}(T_{PDMS}) - P_{atm}(T_{amb}) - P_{sun} - P_{nonrad}(T_{PDMS}, T_{amb}) \quad (1),$$

In equation 1, the power emitted from the PDMS emitter is

$$P_{rad}(T_{PDMS}) = A \int d\Omega \cos(\theta) \int d\lambda I_{BB}(T_{PDMS}, \lambda)\varepsilon_{rad}(\lambda) \quad (S1),$$

Here $\int d\Omega$ is the angular integral of the emitting surface over the hemisphere, $I_{BB}(T,\lambda) = \frac{2hc^2}{\lambda^5}\frac{1}{\exp\left(\frac{hc}{\lambda k_B T}\right)-1}$ is the spectral radiance of a blackbody at a temperature T, h is Planck's constant, k_B is the Boltzmann constant, c is the speed of light, λ is the wavelength, and A is the area of the emitter. ε_{rad} is the spectral emissivity of the emitter. Using this equation, the emissivity of the porous and pristine PDMS emitters can be converted from measured absorption in Figure 2d and 2e, following Kirchhoff's radiation law.

The absorbed atmospheric radiation from the emitter is

$$P_{atm}(T_{amb}) = A\int d\Omega\cos(\theta)\int d\lambda I_{BB}(T_{amb},\lambda)\varepsilon_{rad}(\lambda)\varepsilon_{atm}(\theta,\lambda) \quad \text{(S2).}$$

Here the angle-dependent emissivity of atmospheric $\varepsilon_{atm}(\theta,\lambda)$ is given by $\varepsilon_{atm}(\gamma) = 1 - [t_{atm}(0)]^{\frac{1}{\cos\gamma}}$, where the atmospheric transmittance in zenith direction $t_{atm}(0)$ is estimated by $t_{atm}(0) = 1 - \varepsilon_{atm}(0)$. In this estimation, we modeled the atmospheric emittance in MODTRAN (using the atmosphere model: mid-latitude summer) [R4].

The absorbed solar irradiation is

$$P_{sun} = A\int d\lambda I_{AM1.5}(\lambda)\varepsilon_{rad}(\lambda) \quad \text{(S3).}$$

Here $I_{AM1.5}(\lambda)$ is the AM1.5 Global tilt solar illumination with an irradiance of 1000 W/m^2. The solar irradiance (i.e. the orange spectrum in Fig. 2a) was obtained from national renewable energy laboratory [R5].

The non-radiative heat loss due to heat conduction and convection is

$$P_{nonrad}(T_{rad},T_{amb}) = Aq(T_{rad} - T_{amb}) \quad \text{(S4).}$$

Using these equations, we then estimated the net cooling powers of the porous and pristine PDMS emitters, respectively, as shown in Figure 4e.

Note S4. Estimation of heat fluxes through different roofing materials

The heat flux through different roofing materials was estimated using Fourier's Law ($Q = \frac{-kA}{L}\frac{dT}{dx}$), where Q is the heat flux through the plane, k is the thermal conductivity of the material, L is the thickness of the plane, and A is the plane area. In this estimation, we assumed a linear temperature profile within the single-layered roofing material. The thermal conductivity of the asphalt shingle used in this estimation is 0.5 W/(m·K) [R6]. To estimate the heat flux through the roof in outdoor environment, we employed a hot plate to heat one side of the roofing material to the temperature observed in Figure 5d of the main text, and measured the temperature on the other side. As a result, the temperature difference between the two sides is 7.6 °C for the commercial shingle, 4.6 °C for the white-painted shingle and 6.4 °C for the porous PDMS sponge, respectively. Therefore, the corresponding heat fluxes through the commercial single, white-painted shingle and porous PDMS sponge are 760 W/m^2, 460 W/m^2 and 76 W/m^2, respectively.

Reference

[R1] X. Zhang, J. Qiu, X. Li, J. Zhao, L. Liu. Complex refractive indices measurements of polymers in visible and near-infrared bands, *Appl. Opt.* **2020**, 59, 2337-2344.

[R2] X. Zhang, J. Qiu, J. Zhao, X. Li, L. Liu. Complex refractive indices measurements of polymers in infrared bands, J. Quant. Spectrosc. *Radiat. Transf.* **2020**, 252, 107063.

[R3] Radiative cooling, https://www.comsol.com/model/radiative-cooling-75021, accessed May 2021.

[R4] MODTRAN, http://modtran.spectral.com/modtran_home, accessed May 2021.

[R5] Reference Air Mass 1.5 Spectra, https://www.nrel.gov/grid/solar-resource/spectra-am1.5.html, accessed: July 2021.

[R6] Table of thermal conductivity, specific heat capacity and density, https://help.iesve.com/ve2018/table_6_thermal_conductivity__specific_heat_capacity_and_density.htm, accessed May 2021.
